# Viability and Burden of *Leishmania* in Extralesional Sites during Human Dermal Leishmaniasis

**DOI:** 10.1371/journal.pntd.0000819

**Published:** 2010-09-14

**Authors:** Ibeth Romero, Jair Téllez, Yazmín Suárez, Maria Cardona, Roger Figueroa, Adrian Zelazny, Nancy Gore Saravia

**Affiliations:** 1 Centro Internacional de Entrenamiento e Investigaciones Médicas (CIDEIM), Cali, Colombia; 2 Microbiology Service, Department of Laboratory Medicine, Clinical Center, National Institutes of Health, Bethesda, Maryland, United States of America; Institut Pasteur, France

## Abstract

**Background:**

The clinical and epidemiological significance of *Leishmania* DNA in extralesional sites is obscured by uncertainty of whether the DNA derives from viable parasites. To examine dissemination of *Leishmania* during active disease and the potential participation of human infection in transmission, *Leishmania* 7SLRNA was exploited to establish viability and estimate parasite burden in extralesional sites of dermal leishmaniasis patients.

**Methods:**

The feasibility of discriminating parasite viability by PCR of *Leishmania* 7SLRNA was evaluated in relation with luciferase activity of *luc* transfected intracellular amastigotes in dose-response assays of Glucantime cytotoxicity. Monocytes, tonsil swabs, aspirates of normal skin and lesions of 28 cutaneous and 2 mucocutaneous leishmaniasis patients were screened by kDNA amplification/Southern blot. Positive samples were analyzed by quantitative PCR of *Leishmania* 7SLRNA genes and transcripts.

**Results:**

7SLRNA amplification coincided with luciferase activity, confirming discrimination of parasite viability. Of 22 patients presenting kDNA in extralesional samples, *Leishmania* 7SLRNA genes or transcripts were detected in one or more kDNA positive samples in 100% and 73% of patients, respectively. Gene and transcript copy number amplified from extralesional tissues were comparable to lesions. 7SLRNA transcripts were detected in 13/19 (68%) monocyte samples, 5/12 (42%) tonsil swabs, 4/11 (36%) normal skin aspirates, and 22/25 (88%) lesions; genes were quantifiable in 15/19 (79%) monocyte samples, 12/13 (92%) tonsil swabs, 8/11 (73%) normal skin aspirates.

**Conclusion:**

Viable parasites are present in extralesional sites, including blood monocytes, tonsils and normal skin of dermal leishmaniasis patients. *Leishmania* 7SLRNA is an informative target for clinical and epidemiologic investigations of human leishmaniasis.

## Author Summary

Understanding of the dynamics and distribution of *Leishmania* in the human host is fundamental to the targeting of control measures and their evaluation. Amplification of parasite gene sequences in clinical samples from cutaneous leishmaniasis patients has provided evidence of *Leishmania* in blood, other tissues and sites distinct from the lesion and of persistence of infection after clinical resolution of disease. However, there is uncertainty about the interpretation of the presence of *Leishmania* DNA as indicative of viable parasites. Because RNA is short-lived and labile, its presence provides an indicator of viability. We amplified *Leishmania* 7SLRNA, a molecule involved in intracellular protein translocation, to establish viability and estimate parasite load in blood monocytes, tonsil swab samples, and tissue fluid from healthy skin of patients with dermal leishmaniasis. Results showed that during active dermal leishmaniasis, viable *Leishmania* are present in blood monocytes, tonsils and normal skin in quantities similar to that in lesions, demonstrating widespread dissemination of infection and subclinical involvement of tissues beyond the lesion site. *Leishmania* 7SLRNA will be useful in deciphering the role of human infection in transmission.

## Introduction

Prevention and control of dermal leishmaniasis in the New World continues to elude measures based on case identification and treatment. Persistent infection [1] has favored the development of secondary resistance and relapse following treatment and resolution of lesions. Moreover, primary resistance [2] supports the dissemination of tolerant or resistant organisms via anthroponotic transmission.

Understanding of the dynamics and distribution of *Leishmania* in the human host is fundamental to the targeting of control measures and indeed to their evaluation. The ability to detect and quantify live *Leishmania* using molecular tools would allow crucial gaps in the natural history of human infection with *Leishmania* of the *Viannia* subgenus to be addressed.

Diverse genetic targets and amplification methods have been utilized for the detection, identification and quantification of *Leishmania* in lesions and other tissues [3], [4], [5]. The persistence of *Leishmania* infection following resolution of disease has been supported by serologic, immunohistochemical and molecular evidence. *Leishmania* kDNA has been the principal molecular target, yielding evidence of parasites in extralesional tissues including scars, normal skin, and blood monocytes [5], [6], [7]. However, the high stability of DNA molecules and the possibility of its persistence following parasite death has raised questions concerning the interpretation of its amplification from blood and apparently healthy tissues as indicative of the presence of viable *Leishmania* [8].

Because of its short half life and lability, RNA has been considered a plausible indicator of viability and a diagnostic target for diverse microbial infections [9]. Hence methods such as quantitative nucleic acid sequence-based amplification (QT-NASBA) and real time PCR have been developed and used to detect RNA of microbial pathogens [3], [10], [11]. The 18S ribosomal gene, as well as its abundant RNA transcripts, have been used to detect infection and to estimate parasite burden in leishmanial lesions [10]. However, *Leishmania* RNA targets have yet to be exploited to establish parasite viability or the presence of parasites in extralesional tissues.

7SLRNA is an integral component of the signal recognition particle, a ribonucleoprotein complex of 6 polypeptides and RNA that mediates protein translocation across the endoplasmic reticulum. The genetic divergence of mammalian and trypanosomatid 7SLRNA, its abundance in the cytoplasm [11], [12], and short half life support the feasibility of sensitive and specific detection of live parasites by amplification of this target.

We show that 7SLRNA transcript copy number is proportional to the number of live parasites and demonstrate the presence of *Leishmania* 7SLRNA genes and transcripts in blood monocytes, normal skin and tonsilar mucosa of patients with dermal leishmaniasis.

## Materials and Methods

### Ethics statement

The study protocol, informed consent and sampling procedures were approved by the CIDEIM institutional review board for studies involving human subjects and conducted in compliance with national and international guidelines for the protection of human subjects from research risks. Written informed consent was obtained from all participants.

### Parasites

Promastigotes of *L. (Viannia) panamensis* (MHCOM/CO/86/1166) transfected with the firefly luciferase (LUC) (*L. panamensis-*LUC) reporter gene using the pGL2-α-NEOαLUC expression vector essentially as described [13] were cultured in Schneiders' Drosophila medium (Sigma) [14].

### Patients

Thirty patients with parasitologically confirmed active tegumentary leishmaniasis were included in the study. Participants in this study were patients consulting at Centro Internacional de Entrenamiento e Investigaciones Medicas (CIDEIM) in Cali, Colombia, and who were included in a prior investigation [5]. Inclusion criteria and clinical diagnosis were as previously described [5].

### Sampling

Blood monocytes, tonsil swabs, healthy skin and lesion aspirates were obtained from patients with active parasitologically confirmed dermal leishmaniasis. Blood monocytes were separated from a 10 mL sample of peripheral blood using the Nycoprep system (1.068 g/mL; Axis Shield) following the manufacturer's protocol. Tonsilar swab samples were obtained by gently rubbing with a swab developed for harvesting DNA samples (BuccalAmp kit; Epicenter Biotechnologies). Dermal tissue fluid samples were obtained by aspiration with a tuberculin syringe from healthy skin sites located at a distance of 8–10 cm from cutaneous lesions in the direction of lymphatic drainage, and from the lesion. All samples were stored in TRIzol at -70°C until processing.

### Sample processing

RNA and DNA were extracted using the AllPrep DNA/RNA Minikit (Qiagen) in accordance with the manufacturer's instructions. DNA was used for PCR/Southern blot and quantitative real-time PCR (qPCR) assays. Total DNAse I-treated (Qiagen) RNA was used for quantitative real-time reverse transcriptase PCR (RT- qPCR).

*L. Viannia* kDNA was amplified from all samples by PCR using the LVB1 primers followed by Southern blot hybridization [5]. kDNA positive samples were then evaluated by real time PCR to detect live parasites and estimate parasite burden using 7SLRNA transcript and gene amplification, respectively. The human GAPDH gene was amplified as loading and inhibition control (gene specific TaqMan assay kits, Applied Biosystems).

### Real time PCR assays

#### Probes and primers

Real-time PCR assays were performed to amplify a 173bp fragment of the 7SLRNA gene and its transcript using the primers TRY7SL.For.1 and TRY7SL.Rev.1 as previously described [11]. Additionally, two specific *Leishmania* 7SLRNA probes that hybridized within the amplification product were used: an “up” FL 3′ labeled 7SL probe hybridized at the 43–65 bp sequence of the 7SLRNA gene (5′ TGC TGC GTT GAC GTG GTG CTC TG 3′), and a “down” LCRED 640 5′labeled 7SL probe hybridized the 67–91 bp sequence (5′ TTG GCT GTG TGT CGG TGT GGC CTG C 3′).

#### RT-qPCR and qPCR conditions

100 ng of total RNA were reverse transcribed using the SuperScript III first-strand synthesis system superMix (Invitrogen). For the PCR reaction, 100 ng of total DNA or 3 µL cDNA were added to the amplification mix containing 4 µL of 5× LightCycler FastStart DNA Master^PLUS^ HybProbe master mix (Roche Applied Science), 1 µM of each primer, 0.25 µM of each FL - LCRED640 probe, and completed to a final volume of 20 µL with ultra pure water.

Amplification and real-time measurements were performed with a LightCycler (Roche Applied Science) under the following conditions: 95°C for 6 min, followed by 50 PCR amplification cycles consisting of 5 s at 95°C, 10 s at 60°C, 5 s at 72°C. In the final cycle, the melting curve was obtained by heating at 95°C for 30 s and subsequently cooling samples at 40°C for 15 s. Each PCR run included negative controls for PCR reagents, reverse transcription and sample extraction. The threshold cycle value (Ct) was calculated for each sample automatically by the LightCycler 2.0 software version 4.1 (Roche Applied Science). A standard curve was obtained using 10-fold serial dilutions of 7SLRNA gene cloned in pCR2.1 TOPO TA vector. Ct values were plotted against standards of known 7SLRNA plasmid concentration ranging from 10^7^ to 10^2^copies/reaction, and the number of 7SLRNA copies in the samples was calculated from the standard curve.

### Cytotoxicity assay and evaluation of viability of intracellular amastigotes

Phorbol myristate acetate (PMA)-differentiated human U937 macrophages were exposed to human AB+ serum opsonized antimony sensitive *L. panamensis-*LUC promastigotes for 2 h then washed to remove extracellular parasites. Cultures were treated with pentavalent antimony (Sb^v^) over a dose range from 2 to 32 µg/mL for 72 hr at 34°C, 5% CO_2_. Drug-free control was included in all assays. Surviving intracellular *L. panamensis-*LUC amastigotes were detected by luminometry, measuring luciferase activity as an indicator of viability [15]. In parallel, identically infected and drug-treated cells were collected and stored in TRIzol (Invitrogen) for evaluation of parasite viability by RT-qPCR of the 7SLRNA transcripts.

### Statistical analysis

Intra- and interassay variations of quantitative data for the standard curve were measured as coefficients of variation (CV) for each input concentration, calculated as the standard deviation/average ×100% for each triplicate (intra-assay variation) and for all assay output data (interassay variation). Correlation coefficients (r^2^ values) were calculated for each standard curve by linear regression analysis. Amplification efficiency of real-time assays was calculated as E = 10^−1/slope^, where the slope is obtained from the linear regression of the standard curve. Differences in copy number between RT-qPCR and qPCR were analyzed using the Kruskal-Wallis and Mann-Whitney non-parametric test and significant differences were considered at *p*<0.05. Correlation analyses of luminometry vs. RT-qPCR of 7SLRNA for evaluation of parasite viability during drug dose response assays were conducted using the Spearman coefficient.

## Results

### Sensitivity and specificity of the real-time PCR assay

**Figure 1:**
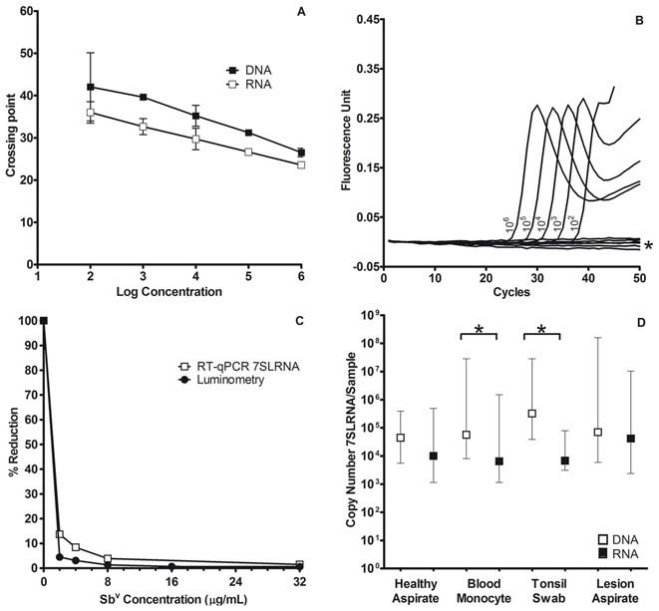
Detection of live *Leishmania* by amplification of 7SLRNA. A: Comparison of Crossing point for DNA and RNA recovered from ten fold dilutions of promastigotes 1×10^6^–1×10^2^ (performed in duplicate), error bars correspond to 2 standard deviations; B: Sensitivity and specificity of *Leishmania (Viannia) panamensis* 7SLRNA real time PCR demonstrated by Amplification Plot of parasite dilutions and negative controls indicated by asterix (PCR without cDNA, Extraction and Retro-transcription negative controls, blood monocytes from healthy donor, *T. cruzi* cDNA); C: Dose response of intracellular *Leishmania (Viannia) panamensis* amastigotes transfected with pGL2-αNEO-αLUC vector to increasing concentrations of Sb^V^. Open squares, percent reduction of 7SLRNA transcript copy number determined by RT-qPCR, filled circles, percent reduction of light emission measured by luminometry; D: Amplification of the 7SLRNA gene and transcripts from different tissue samples of patients with active cutaneous leishmaniasis. Open and filled squares correspond to median +/− min and max RNA and DNA copy number of 7SLRNA respectively. Significant differences are indicated with asterisk, p = 0.02. Healthy skin aspirate refers to unaffected skin 8–10 cm distant from an active lesion.

RT-qPCR and qPCR amplification of total RNA and DNA from serial dilutions of *L. panamensis* promastigotes, revealed a lower detection limit of 10^2^ parasites. As expected, the number of copies of 7SLRNA transcripts in live culture-derived parasites was consistently higher than the number of copies amplified from the corresponding gene (Figure 1A). To determine the specificity of the assay, real-time PCR was performed with RNA and DNA extracted from *L. panamensis*, *Trypanosoma cruzi*, *Mycobacterium tuberculosis,* and human blood monocytes. The *Leishmania* 7SLRNA gene and RNA transcripts were amplified only from *Leishmania* (Figure 1B).

The standard curve of the 7SLRNA gene cloned in pCR2.1 TOPO TA vector for the quantitative assay was linear over concentrations of 10^2^ to 10^7^ copies with an r^2^ of 0.998 and efficiency of 1.977.

### 7SLRNA is amplified from live *Leishmania*

Amplification of *Leishmania* 7SLRNA from infected U937 macrophages was inversely related to antimony concentration and correlated with luciferase activity (Spearman coefficient = 0.914, p = 0.01) in antimony sensitive *L panamensis-*LUC. 7SLRNA copy number was proportional to the number of live parasites based on light emission resulting from luciferase activity in the dose response cytotoxicity assay (Figure 1C), confirming the capacity to quantify live parasites using this RNA target. However 7SLRNA molecules could still be detected, although at a negligible level, at 32 µg/ml Sb^V^, a concentration at which the readout by luminometry approached zero.

### PCR of kDNA and Southern blot analysis

*Leishmania* kDNA was detected by PCR and/or Southern blot in monocytes, tonsil swabs or normal skin of 22 (73%) of the 30 patients. Blood monocytes were the most frequently positive tissue: 19/28 (68%) followed by tonsil swab 13/28 (46%), and normal skin aspirate 11/30 (37%). Lesion aspirates were positive in 26/30 (87%) of patients (Table 1).

**Table 1:** Frequency of detection of parasite kDNA, and 7SLRNA genes and transcripts in blood monocytes, healthy skin aspirate, tonsil swab and lesion aspirates from patients with active cutaneous leishmaniasis.

Sample	Frequency (% positive)		
	kDNA	7SL RNA	
	PCR/Southern Blot	qPCR	RT-qPCR
Blood	19/28 (68)	15/19 (79)	13/19 (68)
Tonsil Swab	13/28 (46)^a^	12/13 (92)	5/12^b^ (42)
Healthy Skin Aspirate^c^	11/30 (37)	8/11 (73)	4/11 (36)
Lesion Aspirate	26/30 (87)	24/26 (92)	22/25 (88)^b^

a Data from Figueroa *et al*, 2009.

b RNA was not detected in one sample.

c Unaffected skin site 8–10 cm distant from active lesion.

### Detection of viable parasites in extralesional sites confirmed kDNA evidence of infection

7SLRNA gene amplification revealed the presence of *Leishmania* in one or more extralesional tissues in 100% (22/22) of patients presenting kDNA in these tissues. Amplification of RNA transcripts confirmed the viability of parasites in one or more extralesional samples in 16/22 (73%) of these patients. 7SLRNA transcripts were most frequently detected in monocytes 13/19 (68%), followed by tonsil swab 5/12 (42%) and healthy skin aspirates 4/11 (36%). Of 25 lesion aspirates that were positive for kDNA, 22 (88%) contained detectable 7SLRNA transcripts (Table 1). Human GAPDH was amplified from all samples from which *Leishmania* 7SLRNA genes were not amplified.

### Relative parasite burden in different sites and tissues

In contrast with the higher amplification of 7SLRNA transcripts versus gene copies in cultured promastigotes, in tissue samples, a higher number of copies of the 7SLRNA gene were amplified than RNA transcripts. This difference was significant (*P* = 0.002) for blood monocytes and tonsil swabs (Figure 1D). Copy number varied from 10^3^ to 10^7^ for both the 7SLRNA gene and transcripts. Notably, copy number of either the 7SLRNA gene or its transcripts did not differ significantly among the extralesional tissues examined or compared with lesions (Figure 1D). Nevertheless, among kDNA positive extralesional samples, the 7SLRNA gene was most frequently amplified from tonsil swabs 12/13 (92%), followed by blood monocytes 15/19 (79%) and healthy skin aspirate 8/11 (73%). The 7SLRNA gene was amplified from of 24/26 (92%) kDNA positive lesion aspirates (Table 1).

## Discussion

This study has shown 7SLRNA transcript copy number to be correlated with the number of viable *Leishmania* and substantiated the presence of live parasites in extralesional tissues of patients with active dermal leishmaniasis. Because amplification of 7SLRNA transcripts detects the presence of live parasites, this target allows a parasitological diagnosis to be achieved. To our knowledge, the results of this study provide the first demonstration of *Leishmania* viability in clinical samples by molecular means.

The finding of live parasite in the blood, normal skin and mucosa of a high proportion of patients with cutaneous disease and no clinical evidence of mucosal involvement demonstrates that the dissemination of *Leishmania* to tissues without triggering disease is common, and may characterize the natural history of human dermal leishmaniasis attributable to species of the *Viannia* subgenus. Furthermore, the high number of parasites (10^3^–10^7^) in small samples of fluid and cells from apparently normal tissues and blood as well as lesions indicate that the parasite burden in humans with active dermal leishmaniasis is substantial. The development of cutaneous leishmaniasis in subclinically infected military personnel following local trauma [16] suggests that the prevalence of parasites in normal tissues may extend to subclinical infection.

The detection and quantification of parasites is important for determining the status of infection, monitoring of treatment and to resolve gaps in the understanding of the natural history of human infection. Few attempts have been made to investigate the presence of *Leishmania* in extralesional tissues of patients with dermal leishmaniasis [5], [7] and quantitative assessment of parasite burden has focused on active lesions [3], [4], [10]. The frequency of *Leishmania* kDNA in blood monocytes and tissue fluid from normal skin was higher in this study than in a previous study using a different primer combination [7]. The high analytical sensitivity of the LV-B1 primer set [17] is confirmed by these results. In the current study, detection of 7SLRNA gene copies and transcripts in kDNA positive samples confirmed the specificity of kDNA amplification from asymptomatic tissue sites, and the applicability of this target to the detection of *Leishmania* in diverse tissue samples. Since the amplified sequence from the 7SLRNA gene corresponds to a region that has been used to distinguish *Leishmania* species [11] identification of the infecting species may also be achievable.

*Leishmania* ribosomal RNA genes (10^2^) and transcripts (10^4^) are more abundant than 7SLRNA genes (10^1^) or transcripts (10^2^) and could provide a more sensitive target [11], [12], [18]. However the relationship between ribosomal RNA gene transcript copy number and parasite viability has not been established and the high and potentially variable number of gene copies in different species and strains [18], while advantageous for detection, could confound estimation of parasite burden.

In this study the efficiency of recovery of *Leishmania* 7SLRNA transcripts from patient samples was lower than that of the corresponding genomic sequences despite the higher copy number of transcripts observed in cultured promastigotes. The lower recovery from patient samples is consistent with the shorter half life, lability and vulnerability of RNA to degradation *in vivo*, as well as during sample processing [9], [19]. Nevertheless, 7SLRNA transcripts were detected in the majority (64%) of kDNA positive samples. The association of 7SLRNA with six polypeptides in the signal recognition particle may confer stability and favor recovery of this parasite RNA molecule [20].

Because of the persistence of DNA after cell death, RNA targets have been exploited for the assessment of the viability of microbial pathogens [9]. However, the size and the location of the target sequence within a gene transcript can influence the efficiency of detection and correlation of its presence with viability [21]. Although shorter sequences are more sensitive targets for PCR reactions they are also more likely to persist intact in non-viable cells whereas longer sequences are more vulnerable to degradation and hence have been more closely correlated with viability defined by replication in culture [19].

The real time PCR assay used in this study was based on a short hypervariable internal sequence (178 bp) of the 7SLRNA gene flanked by conserved primer binding sites. The relatively short length of the amplified target might have contributed to the residual presence at high drug concentration in vitro. Nevertheless, the correlation of copy number of 7SLRNA transcripts with the dose and time dependent reduction of parasite viability in response to Glucantime substantiates the validity of this target as a marker of viability.

Although *Leishmania* have occasionally been isolated from blood or aspirates from normal skin of patients with dermal leishmaniasis [1], [7], [22], [23], conventional parasitological methods are inefficient and of low sensitivity. Real time amplification of the 7SLRNA gene or transcripts provides a rapid and efficient method for the detection and quantification of viable parasites in tissue samples. Isothermal sequence amplification (QT-NASBA) of 18SRNA has been successfully used to detect and quantify *Leishmania* in lesion biopsies obtained from patients with cutaneous leishmaniasis before and after treatment with a lower limit of detection of 2 parasites [10]. Based on the assumption that approximately 250 7SLRNA transcripts are present per cell [12], the limit of detection of RT-qPCR of 7SLRNA, according to the plasmid-derived standard curve, was 10 parasites in lesion aspirates and 5 parasites in blood monocytes. Despite its higher sensitivity compared to Real Time PCR, QT-NASBA is a more complex and demanding method [24].

In conclusion, *Leishmania* viability, parasite burden and infection of extralesional tissues can be parasitologically established by amplification of 7SLRNA genes and transcripts. During active dermal leishmaniasis, viable *Leishmania* are present in blood monocytes, tonsils and normal skin, demonstrating dissemination of infection without triggering pathogenesis. 7SLRNA genes and transcripts are informative targets for clinical and epidemiologic studies to elucidate the natural history of human leishmaniasis and the participation of human infection in transmission, and may also be useful in the evaluation of immunoprophylaxis and other preventive intervention strategies.
